# Identification and validation of a novel glycolysis-related gene signature for predicting the prognosis in ovarian cancer

**DOI:** 10.1186/s12935-021-02045-0

**Published:** 2021-07-06

**Authors:** Jing Yu, Ting-Ting Liu, Lei-Lei Liang, Jing Liu, Hong-Qing Cai, Jia Zeng, Tian-Tian Wang, Jian Li, Lin Xiu, Ning Li, Ling-Ying Wu

**Affiliations:** 1 Department of Gynecologic Oncology, National Cancer Center/National Clinical Research Center for Cancer/Cancer Hospital, Chinese Academy of Medical Sciences and Peking Union Medical College, Beijing, 100021 China; 2 Department of Blood Grouping, Beijing Red Cross Blood Center, Beijing, 100088 China; 3 State Key Laboratory of Molecular Oncology, National Cancer Center/National Clinical Research Center for Cancer/Cancer Hospital, Chinese Academy of Medical Sciences and Peking Union Medical College, Beijing, 100021 China

**Keywords:** OC, Glycolysis, Signature, TCGA, Nomogram, Immune, Prognosis

## Abstract

**Background:**

Ovarian cancer (OC) is the most lethal gynaecological tumor. Changes in glycolysis have been proven to play an important role in OC progression. We aimed to identify a novel glycolysis-related gene signature to better predict the prognosis of patients with OC.

**Methods:**

mRNA and clinical data were obtained from The Cancer Genome Atlas (TCGA), International Cancer Genome Consortium (ICGC) and Genotype Tissue Expression (GTEx) database. The “limma” R package was used to identify glycolysis-related differentially expressed genes (DEGs). Then, a multivariate Cox proportional regression model and survival analysis were used to develop a glycolysis-related gene signature. Furthermore, the TCGA training set was divided into two internal test sets for validation, while the ICGC dataset was used as an external test set. A nomogram was constructed in the training set, and the relative proportions of 22 types of tumor-infiltrating immune cells were evaluated using the “CIBERSORT” R package. The enriched Kyoto Encyclopedia of Genes and Genomes (KEGG) pathways were determined by single-sample gene set enrichment analysis (ssGSEA) with the “GSVA” R package. Finally, the expression and function of the unreported signature genes ISG20 and SEH1L were explored using immunohistochemistry, western blotting, qRT-PCR, proliferation, migration, invasion and xenograft tumor assays.

**Results:**

A five-gene signature comprising ANGPTL4, PYGB, ISG20, SEH1L and IRS2 was constructed. This signature could predict prognosis independent of clinical factors. A nomogram incorporating the signature and three clinical features was constructed, and the calibration plot suggested that the nomogram could accurately predict the survival rate. According to ssGSEA, the signature was associated with KEGG pathways related to axon guidance, mTOR signalling, tight junctions, etc. The proportions of tumor-infiltrating immune cells differed significantly between the high-risk group and the low-risk group. The expression levels of ISG20 and SEH1L were lower in tumor tissues than in normal tissues. Overexpression of ISG20 or SEH1L suppressed the proliferation, migration and invasion of Caov3 cells in vitro and the growth of xenograft tumors in vivo.

**Conclusion:**

Five glycolysis-related genes were identified and incorporated into a novel risk signature that can effectively assess the prognosis and guide the treatment of OC patients.

**Supplementary Information:**

The online version contains supplementary material available at 10.1186/s12935-021-02045-0.

## Background

Ovarian cancer (OC) is the eighth most commonly diagnosed gynaecological malignancy in the world and the eleventh most lethal gynaecological malignancy in China [1, 2]. Although great advancements in its treatments, including surgery, immunotherapy, and PARP inhibitors, have been achieved, the 5-year overall survival (OS) rate of OC is only 45% [3]. Currently, seventy-five percent of OC patients are diagnosed at an advanced stage [4]. Early assessment, which is of profound significance to improve the quality of life and survival rate of OC patients, can be achieved by identifying effective biomarkers of poor prognosis. Glycolysis is a complicated reaction and is the first step in the catabolism of most carbohydrates, the process does not use any molecular oxygen and is a major metabolic pathway [5]. The reprogramming of metabolism is a key hallmark of cancer, and aerobic glycolysis is the main source of energy for cancer cells [6]. Some studies found that tumor tissues metabolized over 10 times more glucose than normal tissues, and lactic acid levels increased by 2 times [7, 8]. The dysregulation of glycolysis has been demonstrated in a variety of tumors, including OC [9–11]. Glycolytic enzymes such as HK2 and PKM2 are elevated in OC cells and influence tumor progression, indicating that they could be potential prognostic markers and therapeutic targets for OC [12, 13]. Oncogenes (such as AKT and mTOR) and tumor suppressors (such as p53) participate in anti-apoptotic and cell survival mechanisms by regulating these enzymes and by regulating the metabolism and growth of cancer cells [14, 15]. Zhao et al. proved that OC cell impose glucose restrictions on T cells and suppress their function, which could provide a new therapeutic strategy [16]. Our study developed a novel gene signature based on the expression of glycolysis-related genes to assess patient prognosis for OC. Further, we explored the function of abnormal expression of those hub genes in OC, which could become potential therapeutic targets in the future.

## Methods

### Data source

Samples from OC patients with complete prognostic data were utilized in this study. Clinical information and mRNA expression data for 375 OC samples from The Cancer Genome Atlas (TCGA) database were downloaded from the University of California, Santa Cruz (UCSC) Xena Browser (http://xena.ucsc.edu/). Data from 93 tumor samples were obtained from the International Cancer Genome Consortium (ICGC) database (https://icgcportal.genomics.cn/releases/current/Projects/OV-AU). In addition, the gene expression profiles for 88 healthy patients were collected from the Genotype Tissue Expression (GTEx) database. Normalized gene expression was measured as fragments per kilobase of transcript per million mapped reads (FPKM), and the values were log2-transformed. TCGA and GTEx mRNA data were merged into one gene expression profile using the comBat algorithm in the “sva” R package to eliminate batch effects. The Voom standardized method was employed to normalize the RNA-seq data in this study.

### Gene set enrichment analysis (GSEA), gene ontology (GO) analysis and Kyoto Encyclopedia of Genes and Genomes (KEGG) pathway enrichment analysis

We searched for the keyword “glycolysis” in Molecular Signatures Database (MSigDB) v4.0 (http://www.broadinstitute.org/gsea/index.jsp), and eight glycolysis-related gene sets (Additional file 1: Table S1) were downloaded to construct a glycolysis gene expression matrix [17]. The “limma” R package was used to identify glycolysis-related differentially expressed genes (DEGs) with a false discovery rate (FDR) < 0.05 between ovarian tumor tissues and normal tissues. GSEA was utilized to assess MSigDB-derived hallmark gene sets to predict the differentially enriched biological processes between normal and OC samples. |Normalized enrichment score (NES)|> 1, nominal (NOM) p-value < 0.05 and FDR q-value < 0.25 were set as the cut-offs. GO analysis and KEGG pathway enrichment analysis were performed with R software via the “clusterprofiler” package.

### Identification and validation of a glycolysis-related gene signature

In the TCGA training set, univariate Cox regression analysis was used to identify the DEGs significantly associated with OS (P < 0.05). Next, multivariate Cox regression analysis using the “forestplot” R package was performed for further screening, and the filtered mRNAs were categorized into risk (hazard ratio (HR) > 1) and protective (0 < HR < 1) types. Based on the multivariate Cox regression analysis results, we developed a prognostic risk score formula. The optimal prognostic mRNA expression levels were multiplied by the relative regression coefficients as follows [18–20]:

$$ {\text{Risk Score}}\left( {{\text{patient}}} \right) = \sum {\text{i}} {\text{ Coefficient }}\left( {{\text{mRNAi}}} \right){\text{ }} \times {\text{ Expression }}\left( {{\text{mRNAi}}} \right) $$

The patients were divided into high-risk and low-risk groups based on the best cut-off risk score [21]. The Kaplan–Meier survival curves, time-dependent receiver operating characteristic (ROC) curve and risk score distribution for OS prediction were assessed to validate the prognostic significance of the risk score.

To further investigate the feasibility of our signature, the TCGA training set was randomly divided into two internal test sets (TCGA, n1 = 188 and n2 = 187), and these datasets and an external test set (ICGC, n3 = 93) were applied to validate our signature. Similar to the training set approach, survival curves for the low-risk and high-risk groups were plotted by the Kaplan–Meier method, and risk score distribution was presented with the “pheatmap” R package in the three test sets.

### Construction of the nomogram

The TCGA training set was used to construct a nomogram for predicting survival outcomes (1-year, 3-year and 5-year survival) via the “rms” packages in R. Calibration plots were drawn to determine the consistency between the actual survival rates and the nomogram-predicted rates.

### Gene set variation analysis (GSVA) and tumor-infiltrating immune cell analysis

To determine the correlation between the risk score and biological pathways, we assessed the corresponding gene expression profiles of these samples via single-sample gene set enrichment analysis (ssGSEA) using the “GSVA” R package. An enrichment score corresponding to each function was obtained for each sample, and the correlation between these functions and the risk score was further calculated.

The RNA-seq data from the TCGA training set were used to calculate the abundance of 22 types of infiltrating immune cells in each sample using the CIBERSORT algorithm. Differences in the abundance of immune cells between the high risk and low risk groups were explored by the “limma” package in R.

### Tissue specimens

Fresh OC tissues and adjacent normal tissues were collected from the Chinese Academy of Medical Sciences and the CAMS & PUMC Medical College. No patients received treatment before surgery, and all patients signed informed consent forms provided by the Cancer Hospital, CAMS & PUMC. The primary tumor area and morphologically normal surgical margin tissue were immediately isolated from each patient by an experienced pathologist and stored in liquid nitrogen until use. The study was approved by the Ethics Committee of the Cancer Institute (Hospital), CAMS & PUMC (17-099/1355).

### Cell culture

The human OC cell line Cavo3 was provided by the Cell Resource Center, IBMS, CAMS/PUMC. The cell lines were cultured in DMEM supplemented with 10% foetal bovine serum (Invitrogen, San Diego, CA) at 37 °C under 5% CO_2_ in a humidified incubator.

### Transfection and lentiviral transduction

All plasmid constructs generated are described in Additional file 1: Table S2. Cells were transfected with overexpression plasmid using Lipofectamine 2000 (Invitrogen) following the manufacturer’s protocols. To generate the lentivirus, 293FT cells (Invitrogen) were co-transfected with psPAX2, pMD2G, pLVX-ISG20-IRES-Neo or pLVX-SEH1L-IRES-Neo. Forty-eight hours after transfection, the lentiviral supernatants were collected and filtered through a 0.45-μm filter. The lentiviruses were added to media containing 8 µg/ml polybrene (Sigma, St. Louis, MO, USA) and transduced into Cavo3 cells according to the manufacturer’s instructions. Stable cell strains expressing ISG20 or SEH1L were selected for at least 1 week using G418 (0.5 mg/ml, Invitrogen).

### Total RNA extraction and quantitative real-time PCR

RNA extraction and quantitative real-time PCR (qRT-PCR) analysis were performed as described previously [22]. The primer sequences are shown in Additional file 1: Table S3. GAPDH served as an internal control.

### Western blotting and immunohistochemistry analysis

Western blotting and immunohistochemistry (IHC) methods were performed as described previously [22]. The IHC quantitation analysis was calculated by ImageJ software and statistically analyzed in three random fields. The antibodies used were as follows: anti-ISG20 antibody (Proteintech, Wuhan, China), anti-SEH1L antibody (Sigma, St. Louis, MO, USA), and anti-ERK1/2 antibody (Cell Signaling Technology, Danvers, MA, USA).

### Cell viability assays

Cells were seeded into 96-well plates at a concentration of 2000 cells per well. Cell viability was detected by Cell Counting Kit-8 assay (CCK-8, Dojindo, Japan) according to the manufacturer’s protocol. The absorbance at 450 nm was measured using an automatic microplate reader (BioTek, Winooski, VT, USA). Measurements were taken every 24 h for 7 consecutive days.

### Cell migration and invasion assays

For the migration assay, 700 μl DMEM with 20% serum was added to the lower chamber of a Transwell plate (Corning, NY, USA), and 1.5 × 10^5^ cells were added to 200 μl serum-free DMEM in the upper chamber. After incubation for 24 h at 37 °C, the Transwell chamber was removed, cleaned once with PBS, and fixed solution (methanol: acetone = 1:1) was fixed for 30 min, then stained with 0.5% crystal violet for 30 min. The chamber was rinsed with PBS, and the cells on the upper side of the filter were carefully wiped away. The relative cell density was measured by ImageJ: saved the results of the migration and invasion experiments at × 50 magnification in PNG format, eliminated the influence of background with ImageJ and randomly selected four fields to get the mean gray value, used the mean gray value to assess Transwell assay. The data were presented as the mean ± SD from three separate experiments. For the invasion assay, the procedure was similar to the migration assay, except the Transwell membrane was coated with 300 ng/ml Matrigel (BD Biosciences, San Jose, CA, USA) and the added cells were incubated for 36 h.

### Xenograft model tumor assay

The animal experiments were approved by the Animal Center of the Institute of National Cancer Center/Cancer Hospital, CAMS & PUMC (NCC2021A002), and followed the National Institutes of Health guide for the care and use of Laboratory animals. Four-week-old female nude mice (BALB/c-nu, HFK Bioscience, Beijing, China) were subcutaneously injected with 3 × 10^6^ Caov3 cells stably expressing pLVX-ISG20-IRES-Neo, pLVX-SEH1L-IRES-Neo or empty vector. The tumor size was measured every 5 days. The tumor volume (V) was calculated with the following formula: V = 0.524 × L × W^2^, where L is the length and W is the width of the tumor. At the end of the experiment (after 3 weeks), the mice were euthanized by an intraperitoneal injection of 100 mg/kg pentobarbital sodium (Sigma, St. Louis, MO, USA), and the xenograft tumors were resected and weighed.

### Statistical analysis

All statistical analyses were performed with R software 3.5.3 and SPSS 22.0 software (SPSS Inc. Chicago, IL, USA). Statistical significance was established at a probability value of P < 0.05. The difference in OS between groups was assessed by Kaplan–Meier analysis with the log-rank test. Student’s t-test was used to determine the significance of differences between two groups, and ANOVA was used for comparisons between more than two groups.

## Results

### Identification and enrichment analysis of glycolysis-related DEGs

**Fig. 1:**
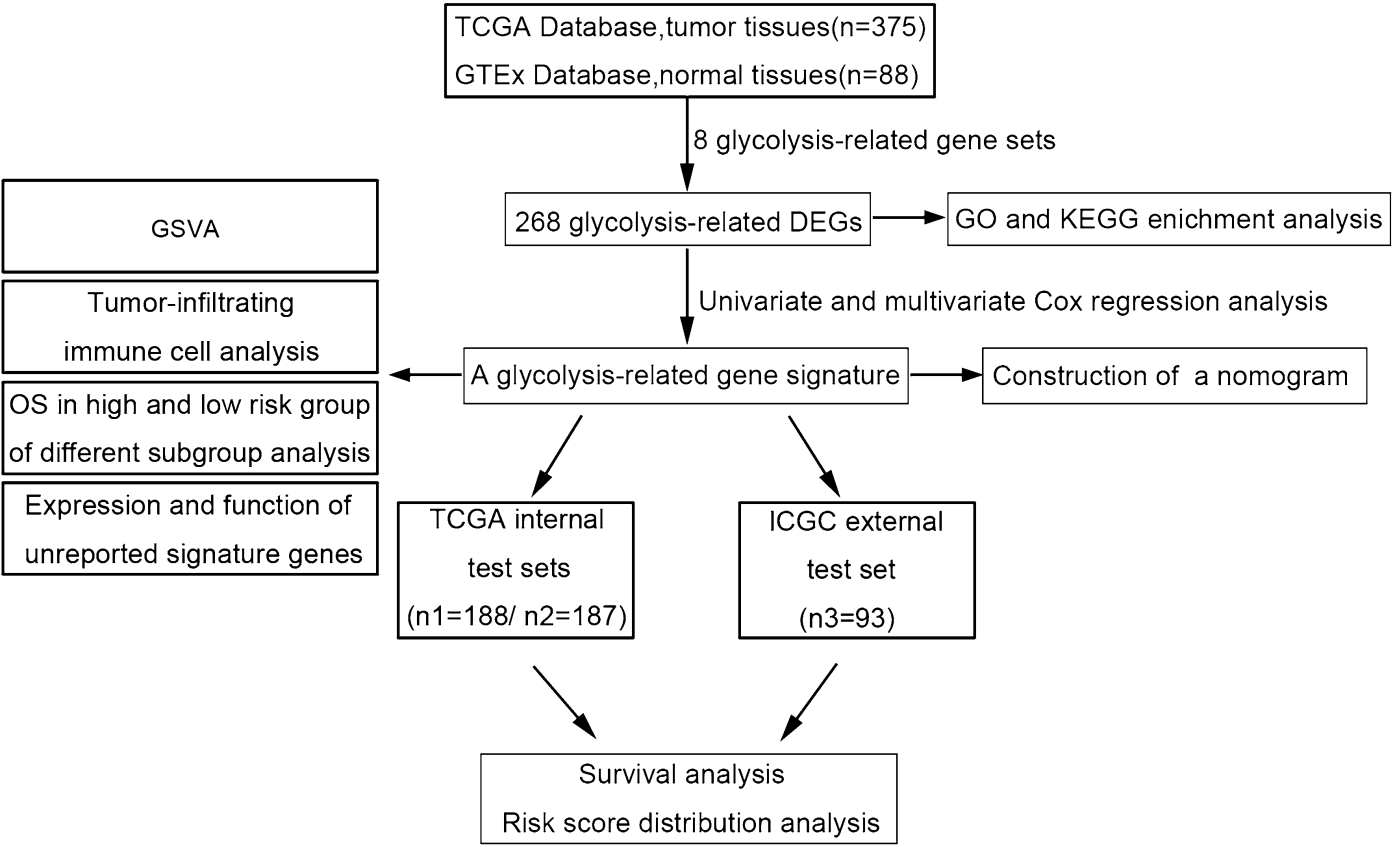
Flow chart for the research

**Fig. 2:**
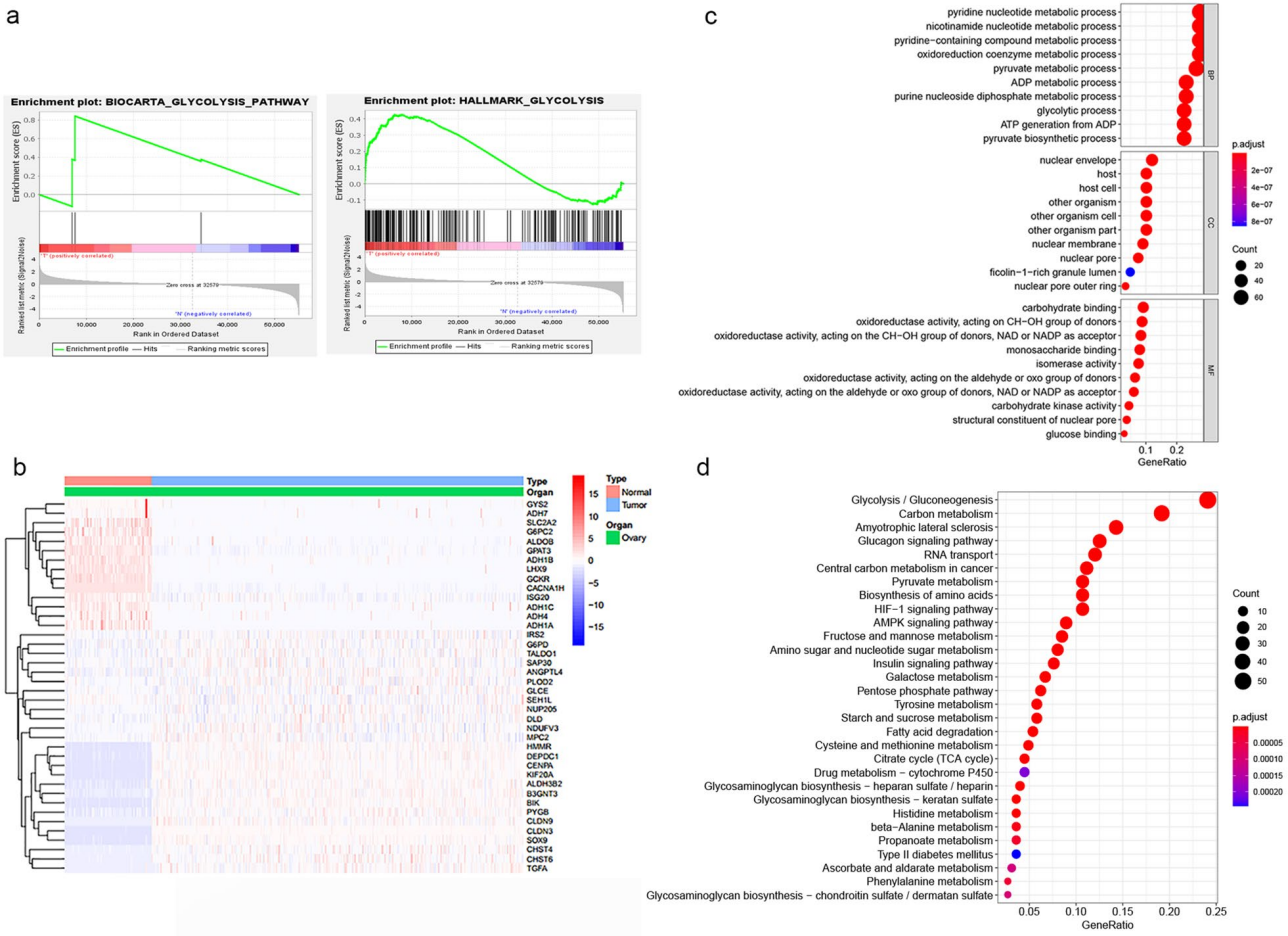
Identification of glycolysis-related DEGs in the TCGA training set and functional analysis of DEGs. **a** GSEA of two gene sets that were significantly differentially enriched between normal and tumor tissues. **b** Heat map of representative DEGs. The colours in the heat map represent the expression levels (blue to red represents the spectrum from low to high. **c** The results of GO enrichment analysis are shown in a bubble chart: molecular function (MF), biological process (BP), and cellular component (CC). **d** KEGG enrichment analysis of DEGs

To describe our research more clearly, we created a flow chart (Fig. 1). Clinical and transcriptome data from 375 patients who provided tumor tissues were downloaded from TCGA database, and the data for 88 normal tissues were obtained from the GTEx database. To identify whether eight glycolysis-related gene sets showed significant differences between normal and ovarian tumor tissues, GSEA was conducted; the results revealed that the BIOCARTA_GLYCOLYSIS_PATHWAY (NES = 1.53, NOM p-value = 0.002 and FDR q-value = 0.001) and HALLMARK_GLYCOLYSIS (NES = 1.8, NOM p-value = 0.0024 and FDR q-value = 0.004) gene sets were significantly differently enriched between the two groups (Fig. 2a), suggesting that glycolysis plays an essential role in OC.

A total of 268 glycolysis-related DEGs, consisting of 149 upregulated and 119 downregulated genes, were observed between tumor tissues and normal tissues. A heat map of representative DEGs is shown in Fig. 2b. Moreover, we performed GO analysis and KEGG pathway enrichment analysis to verify whether the DEGs are involved in glycolysis. The results showed that the enriched biological process (BP) term was related to the glycolytic process, the enriched molecular function (MF) term was related to glucose binding (Fig. 2c), and the enriched KEGG pathways were related to glycolysis/gluconeogenesis and amino sugar and nucleotide sugar metabolism (Fig. 2d), which demonstrated that these DEGs are indeed related to glycolysis.

### Construction and validation of a risk signature with five glycolysis-related genes

**Fig. 3:**
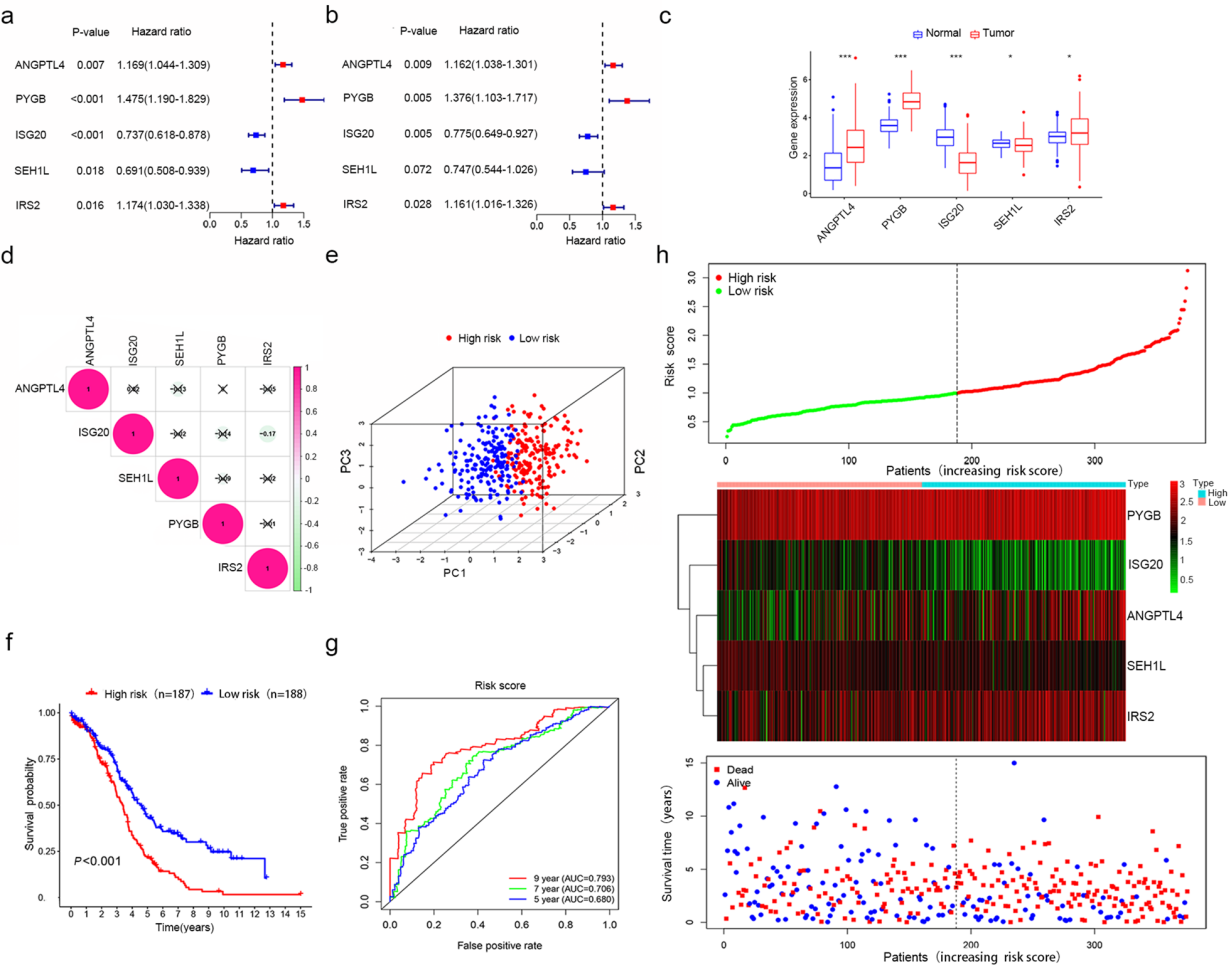
Construction and validation of the risk signature in the TCGA training set. **a**, **b** Five glycolysis-related genes (ANGPTL4, PYGB, IRS2, ISG20 and SEH1L) were selected to construct a risk signature according to univariate Cox regression analysis (**a**) and multivariate Cox regression analysis (**b**). **c** Differential mRNA level between ovarian tumor tissues and normal tissues in TCGA training set. *P < 0.05; **P < 0.01; ***P < 0.001. **d** The correlations between the mRNA levels of the five genes. **e** PCA based on mRNA data between the low-risk (n = 187) and high-risk groups (n = 188). **f** The Kaplan–Meier survival curves of the low-risk group and the high-risk group were distinct, indicating the prognostic value of the risk signature. **g** ROC curves were used to assess the efficiency of the risk signature for predicting 5-, 7- and 9-year survival. **h** The risk score distribution, the expression profiles of the five genes and the survival status of patients

Next, we performed univariate and multivariate Cox regression analyses between the 268 candidate genes above and the survival data. A forest plot of HRs showed that ANGPTL4, PYGB and IRS2 were risk factors (HR > 1), and ISG20 and SEH1L were protective factors (HR < 1) for OS (Fig. 3a and b). The survival analyses of the five genes are presented in Additional file 1: Fig. S1a–e. Ultimately, five glycolysis-related genes, ANGPTL4, PYGB, IRS2, ISG20 and SEH1L, were selected to establish a gene signature.

We searched the cBioPortal website for alterations in the above five gene. Additional file 1: Fig. S2a indicates that the five genes were altered in 51 (12.81%) of the 398 patients; ANGPTL4 and PYGB showed the most diverse alterations, including amplification and missense mutations (Additional file 1: Fig. S2b). The expression levels of ANGPTL4, PYGB and IRS2 were upregulated and those of ISG20 and SEH1L were downregulated in ovarian tumor tissues compared with normal tissues (Fig. 3c). The correlation analysis showed that the absolute value of the R values was less than 0.3, indicating that these five genes are independent of each other (Fig. 3d).

The risk score was calculated as follows: risk score = (0.1500 × mRNA level of ANGPTL4) + (0.3194 × mRNA level of PYGB) + (− 0.2542 × mRNA level of ISG20) + (− 0.2911 × mRNA level of SEH1L) + (0.1492 × mRNA level of IRS2). The patients were stratified into high-risk and low-risk groups according to the best cut-off value of the risk score. Principal component analysis (PCA) based on genome expression data confirmed the significant distribution difference between the two risk groups (Fig. 3e). In addition, patients with a high risk score presented significantly worse OS than those with a low risk score (Fig. 3f, P < 0.001). To investigate the diagnostic accuracy of the glycolysis-related risk signature, the areas under the time-dependent ROC curves (AUCs) were computed. The AUCs of the risk signature for predicting 5-, 7- and 9-year survival were 0.680, 0.706 and 0.793, respectively (Fig. 3g). These results demonstrate that the risk score can effectively predict patient survival rates. Consequently, as the risk score increased, the expression levels of ANGPTL4, PYGB and IRS2 were upregulated, the expression levels of ISG20 and SEH1L were downregulated, and the mortality rate tended to increase (Fig. 3h).

### Internal and external validation of the risk signature

To determine the authenticity of the above prognostic model, a total of 375 OC patients were randomly divided into two internal test sets based on the TCGA training set. In the first internal cohort, samples were divided into a low-risk group (n = 94) and a high-risk group (n = 94). The Kaplan–Meier survival curves suggested that patients with higher risk scores had poorer prognoses than those with lower risk scores (Additional file 1: Fig. S3a). As the risk score increased, the expression levels of ANGPTL4, PYGB and IRS2 increased, the expression levels of ISG20 and SEH1L decreased, and the proportion of patients who died markedly increased in the first internal cohort (Additional file 1: Fig. S3b). The same conclusions were reached in the second internal cohort (Additional file 1: Fig. S3c and d).

**Fig. 4:**
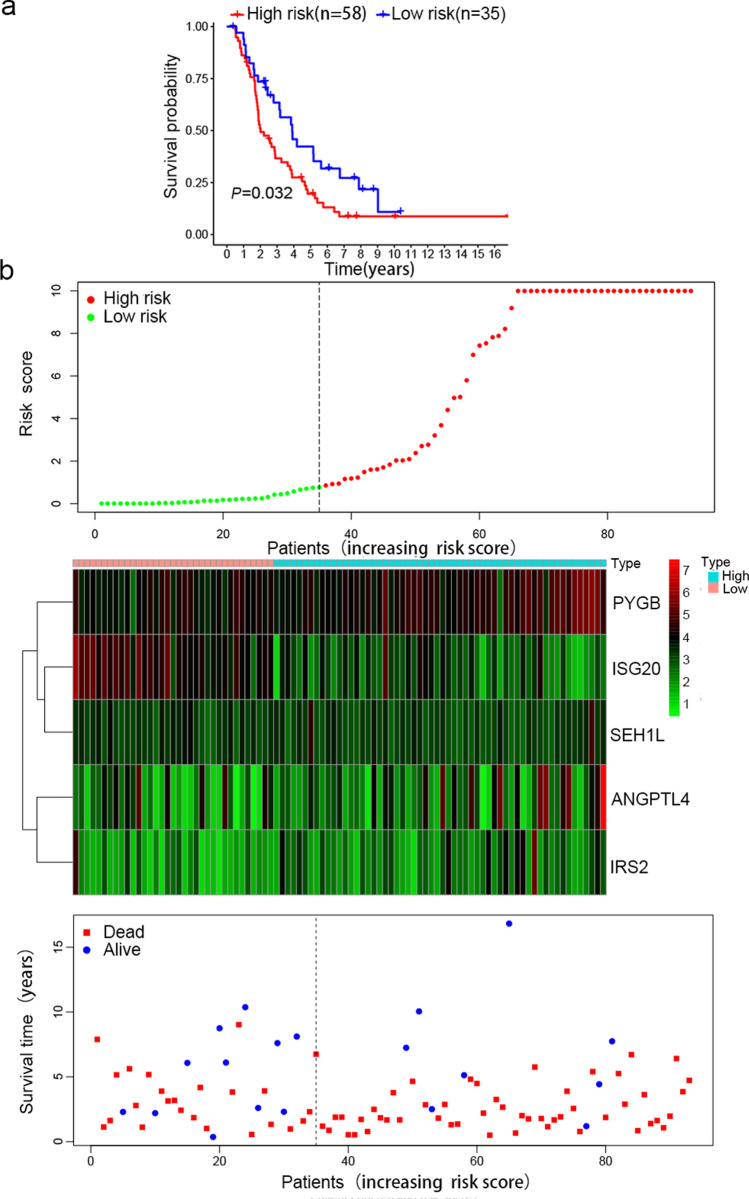
External validation of the risk signature in the ICGC external test set. **a** Kaplan–Meier survival curves showed the prognostic value of the risk signature in the ICGC external test set (n3 = 93). **b** The risk score distribution (When the risk score of the high-risk group was above 10 points, it was recorded as 10, which was convenient for visual display and did not affect the results), the expression profiles of the five genes and the survival status of patients

For the external test set, data from 93 OC samples were collected from the ICGC database, and we further evaluated the performance of the risk signature. The Kaplan–Meier survival curves showed that patients with a higher risk score had a poorer prognosis (Fig. 4a, P < 0.05). In the ICGC test set, as the risk score increased, the expression levels of ANGPTL4, PYGB and IRS2 were upregulated, the expression levels of ISG20 and SEH1L were downregulated, and the number of surviving patients decreased (Fig. 4b). These findings in the ICGC external test set and two internal test sets, which were from different data sources, indicate that the risk signature performs well in predicting the survival of OC patients.

### Assessment of the OS of high-risk and low-risk OC patients in different subgroups

We performed Kaplan–Meier survival analysis according to age, tumor number, tumor status, tumor grade and tumor stage in the TCGA training set. Patients were stratified into the age > 60 or <  = 60 subgroup, tumor number > 20 or <  = 20 subgroup, tumor-free or tumor subgroup, grade 1–2 or grade 3 subgroup, and stage I–II or stage III–IV subgroup. The OS of patients in the high-risk group was significantly shorter than that of patients in the low-risk group in the age > 60 subgroup (Additional file 1: Fig. S4a, P < 0.01), which was consistent with the results in the age <  = 60 subgroup (Additional file 1: Fig. S4b, P < 0.001), tumor number > 20 subgroup (Additional file 1: Fig. S4c, P < 0.05), tumor number <  = 20 subgroup (Additional file 1: Fig. S4d, P < 0.001), tumor-free subgroup (Additional file 1: Fig. S4e, P < 0.05), with-tumor subgroup (Additional file 1: Fig. 4 Sf, P < 0.01), grade 3 subgroup (Additional file 1: Fig. S4h, P < 0.001) and stage III-IV subgroup (Additional file 1: Fig. S4j, P < 0.001). The above findings further showed that our risk signature still has good predictive ability in different clinical subgroups.

### Construction of a nomogram for predicting the 1-, 3- and 5-year survival

**Fig. 5:**
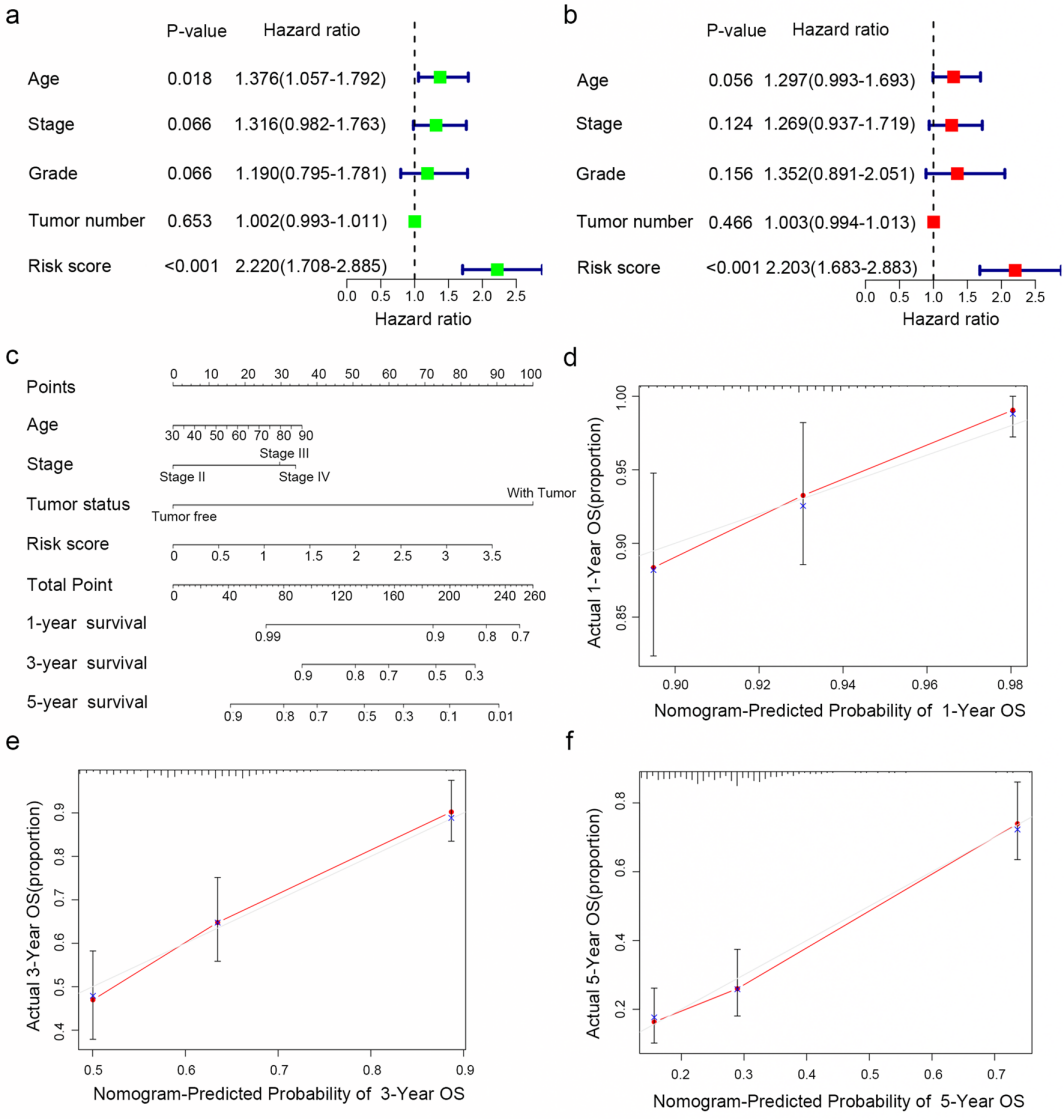
Construction of a nomogram for predicting the 1-, 3- and 5-year survival of OC patients. **a**, **b** Univariate Cox regression analysis (**a**) and multivariate Cox regression analysis (**b**) were applied to evaluate the contribution of each variable to OC survival. **c** A nomogram for predicting the 1-, 3- and 5-year survival rates of OC patients was established. **d**–**f** Calibration curves showed the actual rate versus predicted probability of 1- (**d**), 3- (**e**) and 5-year (**f**) survival

To assess the independent prognostic value of the risk signature, univariate and multivariate Cox regression analyses, including age, stage, grade, tumor number and risk score, were performed in the training set (Fig. 5a and b). The results indicated that the risk score was an independent prognostic factor for OS.

Nomograms can be used to apply risk signatures intuitively and effectively and can be conveniently used to predict outcomes. Age, stage, tumor status and risk score were incorporated into a nomogram. The nomogram predictions for 1-, 3- and 5-year OS are presented in Fig. 5c. As shown in Fig. 5d–f, the calibration curves for the nomogram for 1-, 3- and 5-year survival were almost identical to the standard curve. These results indicate that our nomogram demonstrates fair accuracy for predicting the survival rates of OC patients.

### GSVA and immune infiltration analysis of the risk signature between low- and high-risk group

**Fig. 6:**
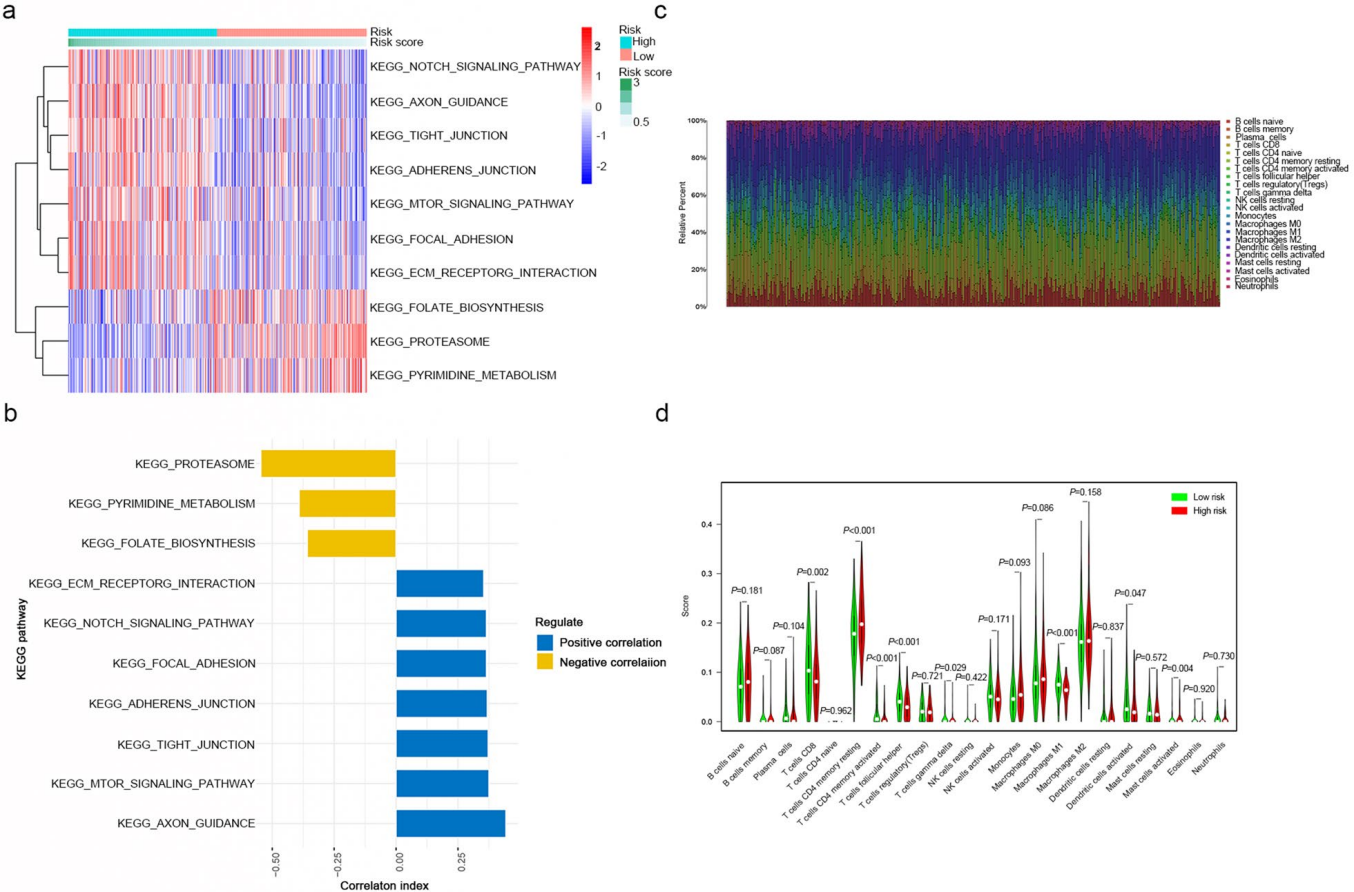
GSVA and immune infiltration analysis of the risk signature between low- and high-risk group. **a** GSVA revealed KEGG pathways associated with the risk signature. **b** The top 10 risk signature-related KEGG pathways. **c** Infiltration abundance of 22 immune cells in each sample; samples were sorted by risk score from left (lowest) to right (highest). **d** The differences in the infiltration level of 22 immune cells between the low-risk and high-risk groups. The green line is the low-risk group, and the red line represents the high-risk group

In order to explore the relationship between the risk score and biological function in different samples, we performed ssGSEA using the “GSVA” R package. Enrichment analysis of risk score groups revealed the top 10 KEGG pathways closely related to the risk score (Fig. 6a). KEGG_AXON_GUIDANCE, KEGG_MTOR_SIGNALING_PATHWAY, KEGG_TIGHT_ JUNCTION, KEGG_ADHERENS_JUNCTION, KEGG_FOCAL_ADHESION, KEGG_ NOTCH_SIGNALING_PATHWAY, and KEGG_ECM_RECEPTOR_INTERACTION were positively correlated with the risk score. KEGG_PROTEASOME, KEGG_PYRIMIDINE_ METABOLISM and KEGG_FOLATE_BIOSYNTHSIS were negatively correlated with the risk score (Fig. 6b).

The mRNA data from the training set were used to determine the infiltration levels of 22 major immune cell types in each sample via the CIBERSORT algorithm (Fig. 6c). As shown in Fig. 6d, the proportions of some infiltrating immune cells were significantly different between the high-risk and low-risk groups. Among them, the infiltration levels of resting memory CD4^+^ T cells, gamma delta T cells and activated mast cells were significantly higher in the high-risk group than in the low-risk group. In contrast, the infiltration levels of CD8^+^ T cells, memory activated CD4^+^ T cells, follicular helper T cells, M1 macrophages and activated dendritic cells were significantly higher in the low-risk group.

### Expression and function analysis of unreported signature genes ISG20 and SEH1L in OC

**Fig. 7:**
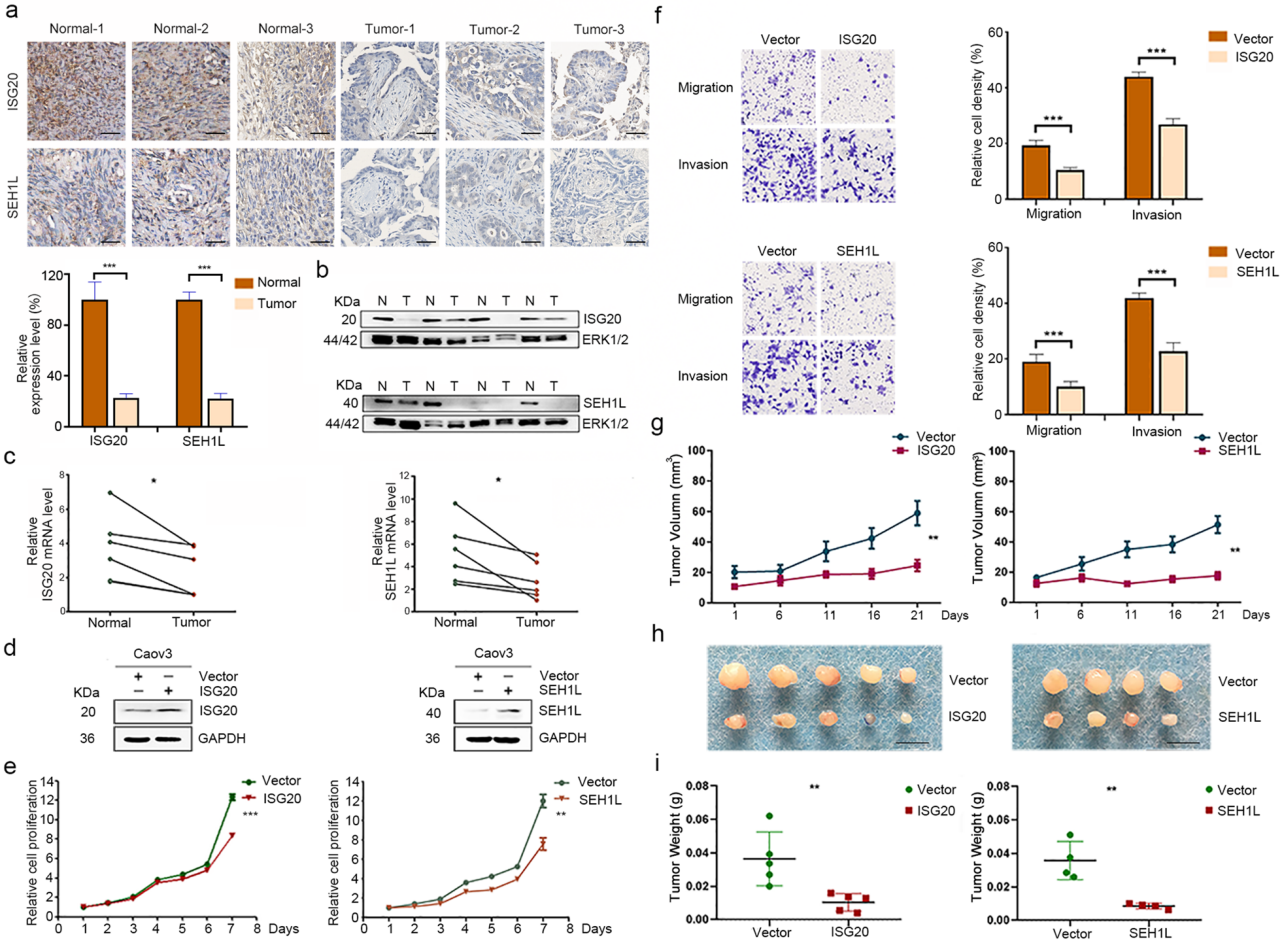
Expression and function analysis of unreported signature genes ISG20 and SEH1L in OC. **a**, **b** Immunohistochemistry (**a**) and western blotting (**b**) analysis of ISG20 and SEH1L protein levels in paired tumor tissues and adjacent normal tissues. Scale bar = 50 μm. **c** qRT-PCR analysis of ISG20 and SEH1L mRNA levels in paired tumor tissues and adjacent normal tissues. *P < 0.05. **d** Western blotting analysis of ISG20 or SEH1L expression in Caov3 cells transfected with pLVX-ISG20-IRES-Neo, pLVX-SEH1L-IRES-Neo or empty vector. **e** Cell proliferation was detected by the CCK-8 assay. **P < 0.01; ***P < 0.001. **f** Cell migration and invasion were evaluated by Transwell assays. **g** Tumor growth curve. h. Xenograft tumors. Scale bar = 1 cm. **i** The weights of xenograft tumors. **P < 0.01; ***P < 0.001

ANGPTL4, PYGB and IRS2 have been reported to be upregulated in OC, and this dysregulation promotes OC cell proliferation, invasion, migration and drug resistance [23–26]. However, the expression and function of ISG20 and SEH1L in OC have not yet been reported. We applied immunohistochemistry, western blotting and qRT-PCR to detect the differences in the expression of ISG20 and SEH1L between paired tumor tissues and adjacent non-tumor tissues. The protein (Fig. 7a, and b) and mRNA (Fig. 7c) levels of both factors were lower in tumor tissues than in normal tissues.

To clarify the functional role of ISG20 and SEH1L in OC cells, overexpression plasmids were constructed and applied to increase their expression levels (Fig. 7d). The CCK-8 assay was applied to detect cell proliferation. The overexpression of ISG20 or SEH1L significantly inhibited the proliferation of Caov3 cells (Fig. 7e). Transwell assays were applied to detect the invasion and migration ability of Caov3 cells in vitro, and the number of cells that passed through the polycarbonate membrane was smaller in the ISG20- or SEH1L-overexpressing group than in the control group, indicating that ISG20 or SEH1L overexpression could significantly inhibit the invasion and migration of Caov3 cells (Fig. 7f).

In addition, nude mice bearing xenograft tumors were divided into the pLVX-ISG20-IRES-Neo, pLVX-SEH1L-IRES-Neo and pLVX-IRES-Neo groups. Stable overexpression of ISG20 or SEH1L by pLVX-ISG20-IRES-Neo or pLVX-SEH1L-IRES-Neo in Caov3 cells reduced tumor growth. The changes in tumor volume and weight in the nude mice are shown in Fig. 7g–i. The results indicated that ISG20 or SEH1L could significantly inhibit tumorigenicity in vivo.

## Discussion

In recent years, research on malignant tumors and energy metabolism has attracted considerable attention. The Warburg effect is illustrated by the metabolic pathway shift from mitochondrial oxidative phosphorylation to glycolysis in tumor cells [27]. Although glycolysis produces ATP less efficiently than aerobic respiration, it satisfies the need for tumor cell proliferation by increasing NADPH synthesis and protects cells from oxidative stress [28]. Despite notable advances in the study of cancer and glycolysis, research on cancer markers associated with glycolysis is limited. In this study, we identified a glycolysis-related gene signature for predicting the prognosis of OC patients.

Despite significant advances in treatment, the OS rate for OC remains poor. There is evidence that clinicopathological parameters (such as tumor-node-metastasis (TNM) stage) are insufficient for accurately predicting patient prognosis [29]. Therefore, a large number of molecules have been examined and identified as biomarkers related to the development and prognosis of cancer. For example, CA125 has been the most widely used biomarker in gynaecological malignancy in the past 20 years, and the detection of mutations in BRCA1 and BRCA2 genes has been widely used in the clinical screening of OC [30, 31]. However, these biomarkers cannot independently predict patient survival. Here, we constructed a gene signature that integrated the prognostic effects of five glycolysis-related genes, and this signature was superior to a single biomarker in predicting disease prognosis.

Consistent with other studies [23–26], we observed that ANGPTL4, PYGB and IRS2 were highly expressed in ovarian tumor tissues and patients with high expression of ANGPTL4, PYGB or IRS2 had significantly lower OS rates than patients with low expression of these factors. ANGPTL4, a secreted glycoprotein in the angiopoietin-like protein family, is involved in glucose and lipid metabolism and also plays an essential role in the prevention of apoptosis, induction of angiogenesis, inhibition of angiogenesis and facilitation of metastasis [23, 32, 33]. It was reported that deregulation of ANGPTL4 slows OC progression through VEGFR2 phosphorylation [26]. Besides, Yang et al. found that the TAZ-ANGPTL4-NOX2 axis regulates ferroptosis and chemotherapy resistance in OC [34]. PYGB is a rate-limiting enzyme in the process of glycogen metabolism that provides energy. Multiple studies have revealed that PYGB is highly expressed in various cancers, including lung cancer, prostate cancer and breast cancer [35–37], and PYGB has also been investigated as a diagnostic molecular marker in gastric cancer [38]. Zhang et al. [39] found that PYGB is upregulated in OC tissue and that overexpression of PYGB is related to a poor prognosis in OC patients. Mechanistically, PYGB promotes OC cell proliferation, invasion and migration via the Wnt/β-catenin signalling pathway [24]. IRS2 plays a critical role in the regulation of the physiological effect of insulin through its specific receptor. Moreover, in terms of the metabolic effect of insulin, high IRS2 expression can promote the reuptake and full utilization of glucose by cells, thus enhancing insulin sensitivity. A previous study indicated that IRS2 is upregulated and activated by the PI3K/AKT pathway to induce the growth and suppress the apoptosis of OC cells [25].

Consistent with the database results, our experiments confirmed that ISG20 and SEH1L are expressed at low levels in tumor tissues and that both of these genes can inhibit the proliferation, invasion, and migration of OS cells. ISG20, a 3′–5′ exonuclease, has been reported to inhibit the replication of some viruses, including hepatitis B virus (HBV), hepatitis C virus (HCV) and human immunodeficiency virus (HIV) [40–42]. Lin et al. [43] found that the overexpression of ISG20 in hepatocellular carcinoma specimens was positively correlated with clinical parameters such as vascular infiltration and tumor size. Moreover, the SEH1L gene encodes part of the NUP107-160 nuclear pore complex, which is involved in mitosis. SEH1L is associated with nail diseases and non-syndromic congenital diseases [44, 45]. Thus far, no study has clarified the mechanisms of ISG20 and SEH1L in OC, so we will design a series of in vivo and in vitro experiments to address this in the future.

In this study, we conducted a comprehensive analysis and constructed a novel gene signature to predict the prognosis of OC patients. The risk signature, based on the expression levels of five-related glycolysis genes, is more economical and clinically feasible than whole-genome sequencing. Nomograms that integrate gene signatures with clinicopathological parameters enable clinicians to more accurately analyse the prognosis of each patient. However, this study also presents several limitations. First, the gene signature needs to be validated further in multi-centre trials and larger patient cohorts because we developed and verified this signature using data from a single centre. Second, BRCA1/2 mutations are important in OC, so we should have factored this into our analyses; we might identify a separate gene signature for patients with BRCA1/2 mutations in the next step.

## Conclusions

Our study identified a novel glycolysis-related signature and established a feasible nomogram model for OS prediction in OC patients that could be applied to guide individualized prognosis evaluation. These signature hub genes in the glycolysis pathway, such as ISG20 and SEH1L, could serve as new treatment targets in the future.

## Supplementary Information

**Additional file 1:** **Table S1.** Eight glycolysis-related gene sets were used to construct a glycolysis gene expression matrix. **Table S2.** Primers for expression vector construction. **Table S3.** Primers for qRT-PCR analysis. **Fig. S1.** Validation of the prognostic value of the five glycolysis related genes. a-e. Kaplan-Meier survival curves of patients stratified by ANGPTL4 (a), PYGB (b), IRS2 (c), ISG20 (d), and SEH1L (e) expression. **Fig. S2.** Gene alteration overview for the five prognostic glycolysis-related genes in 398 OC patients. a. The summary of alterations in the five genes. b. The alterations in each signature gene. **Fig. S3.** Internal validation of the five-gene signature. a, c. Kaplan-Meier survival curves showed the prognostic value of the risk signature in two TCGA internal test sets (n1=188, n2=187). b, d. The risk score distribution, expression profiles of the five genes, and survival status of patients. **Fig. S4.** Assessment of the OS of high-risk and low-risk patients in different subgroups. a. Age>60 subgroup. b. Age<=60 subgroup. c. Tumor number>20 subgroup. d. Tumor number <=20 subgroup. e. Tumor free subgroup. f. With tumor subgroup. g. Grade 1-2 subgroup. h. Grade 3 subgroup. i. Stage I-II subgroup. j. Stage III-IV subgroup.

## Data Availability

The data and materials can be obtained from the first author and corresponding author.

## Abbreviations

OC: Ovarian cancer
DEGs: Differentially expressed genes
GO: Gene ontology
EGG: Kyoto Encyclopedia of Genes and Genomes
GSEA: Gene set enrichment analysis
TCGA: The Cancer Genome Atlas
ICGC: International Cancer Genome Consortium
GTEx: Genotype Tissue Expression
ROC: Receiver operating characteristic
HR: Hazard ratio
OS: Overall survival
