# Supplementary material for: Identification and validation of a novel glycolysis-related gene signature for predicting the prognosis in ovarian cancer
Source: Cancer Cell Int. 2021 Jul 6;21:353. doi: 10.1186/s12935-021-02045-0 (PMC8258938; doi:10.1186/s12935-021-02045-0)
Supplement: Supplementary file 1 — Additional file 1: Table S1. Eight glycolysis-related gene sets were used to construct a glycolysis gene expression matrix. Table S2. Primers for expression vector construction. Table S3. Primers for qRT-PCR analysis. Fig. S1. Validation of the prognostic value of the five glycolysis related genes. a-e. Kaplan-Meier survival curves of patients stratified by ANGPTL4 (a), PYGB (b), IRS2 (c), ISG20 (d), and SEH1L (e) expression. Fig. S2. Gene alteration overview for the five prognostic glycolysis-related genes in 398 OC patients. a. The summary of alterations in the five genes. b. The alterations in each signature gene. Fig. S3. Internal validation of the five-gene signature. a, c. Kaplan-Meier survival curves showed the prognostic value of the risk signature in two TCGA internal test sets (n1=188, n2=187). b, d. The risk score distribution, expression profiles of the five genes, and survival status of patients. Fig. S4. Assessment of the OS of high-risk and low-risk patients in different subgroups. a. Age>60 subgroup. b. Age<=60 subgroup. c. Tumor number>20 subgroup. d. Tumor number <=20 subgroup. e. Tumor free subgroup. f. With tumor subgroup. g. Grade 1-2 subgroup. h. Grade 3 subgroup. i. Stage I-II subgroup. j. Stage III-IV subgroup. [file 12935_2021_2045_MOESM1_ESM.docx]

**Table S1.** Eight glycolysis-related gene sets were used to construct a glycolysis gene expression matrix.

|  | Names of the glycolysis-related genes sets |
| --- | --- |
| 1 | BIOCARTA_GLYCOLYSIS_PATHWAY.gmt |
| 2 | HALLMARK_GLYCOLYSIS.gmt |
| 3 | KEGG_GLYCOLYSIS_GLUCONEOGENESIS.gmt |
| 4 | REACTOME_GLYCOLYSIS.gmt |
| 5 | REACTOME_REGULATION_OF_GLYCOLYSIS_BY_FRUCTOSE_2_6_BISPHOSPHATE_METABOLISM.gmt |
| 6 | WP_COMPUTATIONAL_MODEL_OF_AEROBIC_GLYCOLYSIS.gmt |
| 7 | WP_GLYCOLYSIS_AND_GLUCONEOGENESIS.gmt |
| 8 | WP_HIF1A_AND_PPARG_REGULATION_OF_GLYCOLYSIS.gmt |

**Table S2.** Primers for expression vector construction.

| Plasmid | Primer | Sequence (5’ to 3’) |
| --- | --- | --- |
| pLVX-ISG20-IRES-Neo | Forward | CCGGAATTCATGGCTGGGAGCCGTGAG |
|  | Reverse | CGCGGATCCTCAGTCTGACACAGCCAGGCG |
| pLVX-SEH1L-IRES-Neo | Forward | CTAGTCTAGAATGTTTGTGGCTCGCAG |
|  | Reverse | CGCGGATCCTTAAATCCCTTCATTCTCAGGTAAG |

**Table S3.** Primers for qRT-PCR analysis.

| Gene | Primer | Sequence (5’ to 3’) |
| --- | --- | --- |
| GAPDH | Forward | CAGCCTCAAGATCATCAGCA |
|  | Reverse | TGTGGTCATGAGTCCTTCCA |
| ISG20 | Forward | CTTCCAGGCACTGAAAGAGG |
|  | Reverse | ATCTTCCACCGAGCTGTGTC |
| SEH1L | Forward | GCCACTGGGTTAAAAGGACA |
|  | Reverse | TCTCATGCTGCAAAGACCAC |


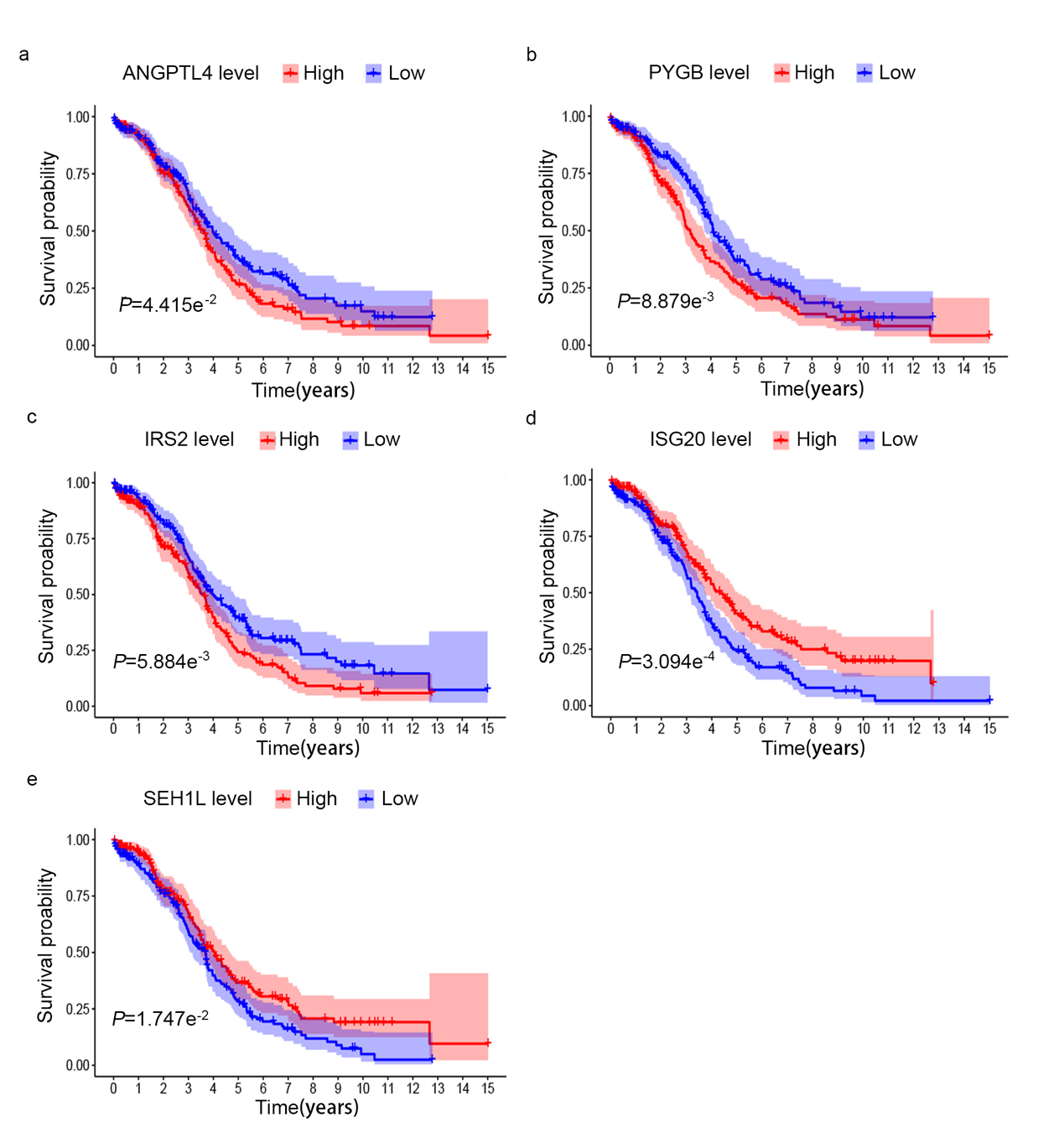


**Fig. S1. Validation of the prognostic value of the five glycolysis‑related genes.** a-e. Kaplan-Meier survival curves of patients stratified by ANGPTL4 (a), PYGB (b), IRS2 (c), ISG20 (d), and SEH1L (e) expression.

**
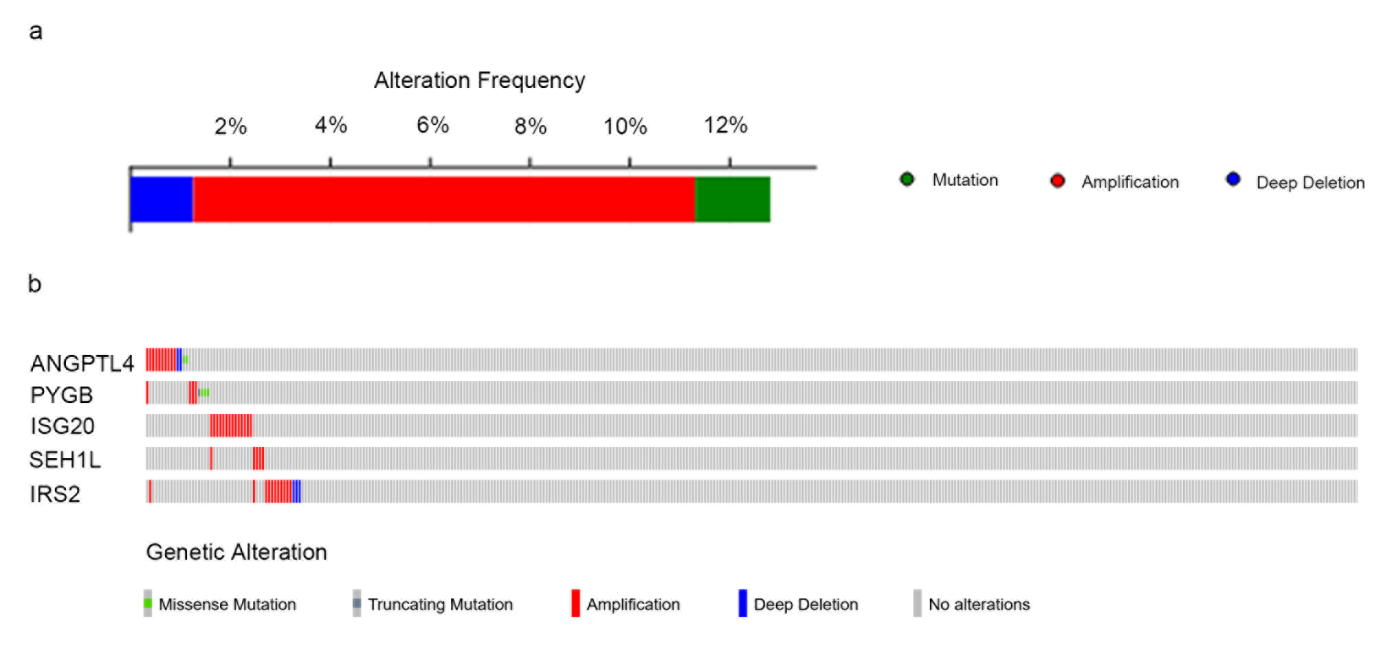
**

**Fig. S2. Gene alteration overview for the five prognostic glycolysis-related genes in 398 OC patients.** a. The summary of alterations in the five genes. b. The alterations in each signature gene.


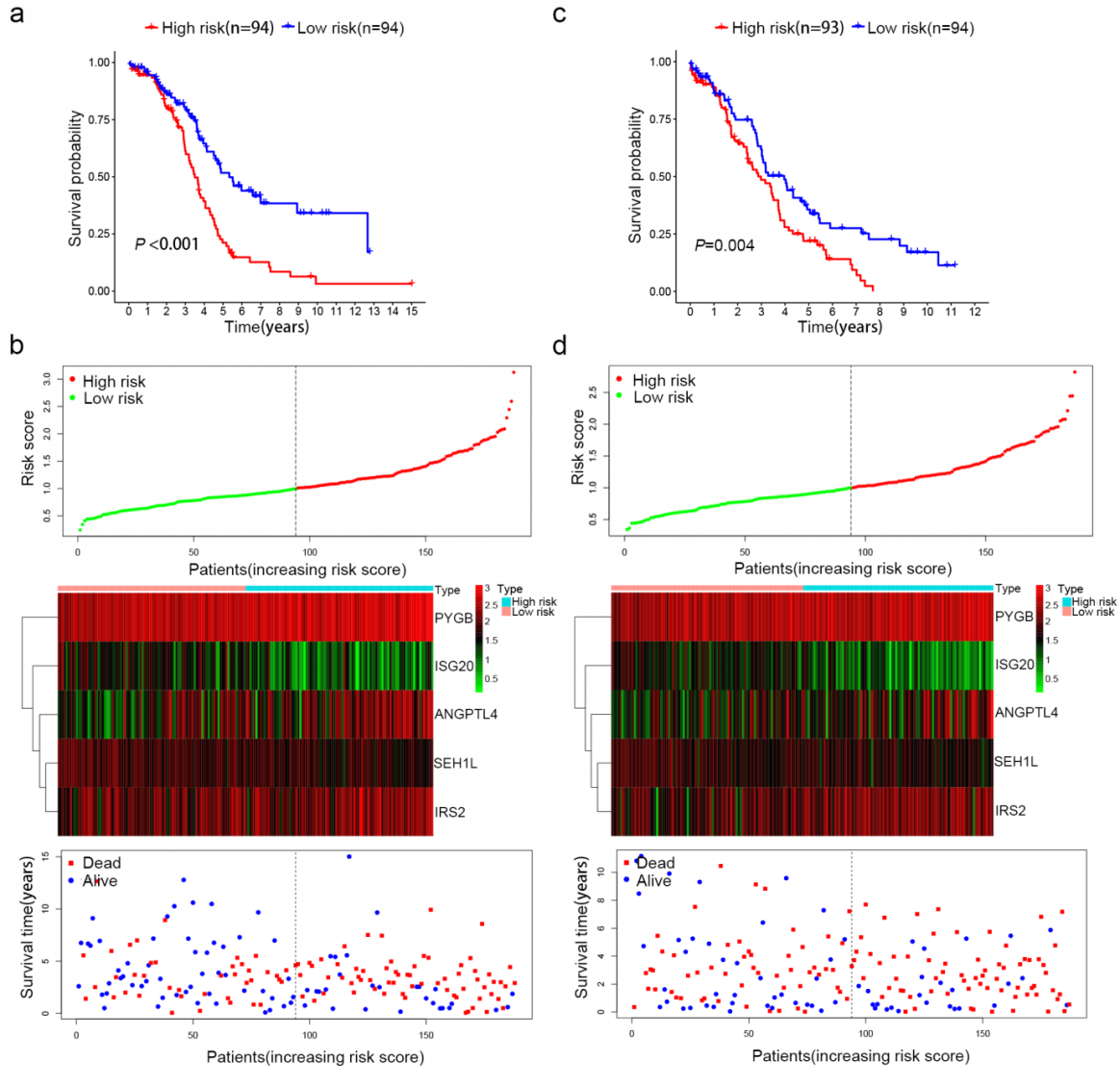


**Fig. S3. Internal validation of the five-gene signature.** a, c. Kaplan-Meier survival curves showed the prognostic value of the risk signature in two TCGA internal test sets (n1=188, n2=187). b, d. The risk score distribution, expression profiles of the five genes, and survival status of patients.


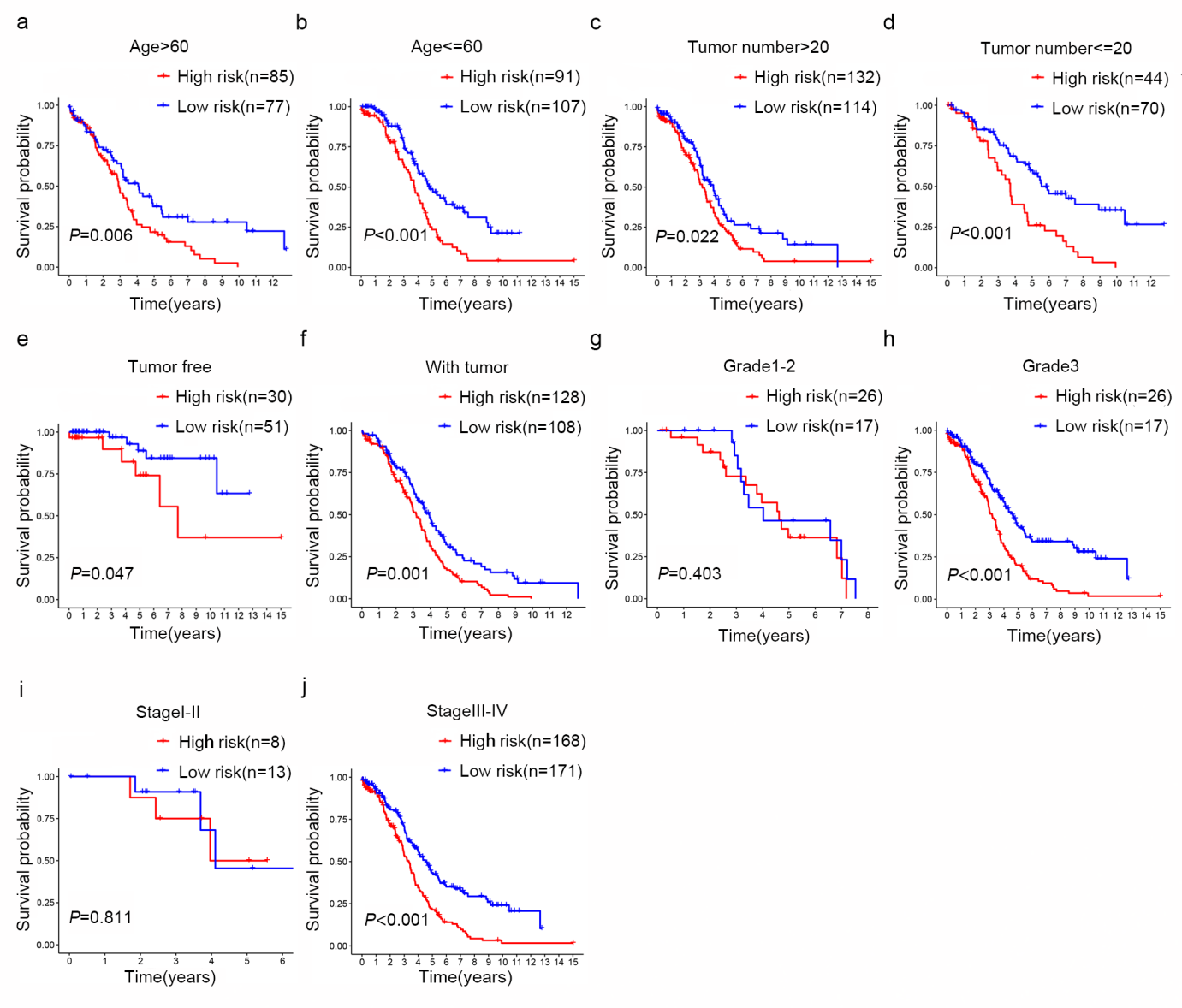


**Fig. S4. Assessment of the OS of high-risk and low-risk patients in different subgroups.** a. Age>60 subgroup. b. Age<=60 subgroup. c. Tumor number>20 subgroup. d. Tumor number <=20 subgroup. e. Tumor free subgroup. f. With tumor subgroup. g. Grade 1-2 subgroup. h. Grade 3 subgroup. i. Stage I-II subgroup. j. Stage III-IV subgroup.
